# Personality Determinants Related to the Use of Selective and Effective Dietary Supplements by Elite Polish Team Sport Athletes

**DOI:** 10.3390/sports12010029

**Published:** 2024-01-12

**Authors:** Maria Gacek, Agnieszka Wojtowicz, Adam Popek

**Affiliations:** 1 Department of Sports Medicine and Human Nutrition, Institute of Biomedical Sciences, University of Physical Education in Kraków, 31-571 Krakow, Poland; 2 Department of Psychology, Institute of Social Sciences, University of Physical Education in Kraków, 31-571 Krakow, Poland; agnieszka.wojtowicz@awf.krakow.pl; 3 Department of Recreation and Biological Renewal, Institute of Recreation and Sports, University of Physical Education in Kraków, 31-571 Krakow, Poland; adam.popek@awf.krakow.pl

**Keywords:** Big Five personality model, ergogenic aids, athletes, team sports

## Abstract

Introduction: The purpose of this research was to analyse relationships between personality traits and the use of selected dietary supplements among Polish athletes training in team sports. This subject matter has not been explored in prior research. Material and Methods: This research was carried out among a group of 213 athletes (men) in the 18–36 age range, with the implementation of a proprietary validated questionnaire for the use of dietary supplements and the NEO-PI-R inventory (Neuroticism–Extraversion–Openness Personality Inventory—Revised). Statistical analyses were performed with the Kruskal–Wallis and Mann–Whitney tests, assuming the following level of significance: α = 0.05. Results: It was shown that athletes who periodically and regularly consumed isotonic drinks, as well as energy bars and gels, were characterised by a lower level of neuroticism than those who did not consume them. Athletes who periodically took multivitamin preparations were characterised by a lower level of extraversion and openness, and those periodically using multimineral preparations were characterised by a higher level of agreeableness than those who did not use these agents. Athletes not taking creatine were characterised by the lowest level of conscientiousness among the study participants. The use of protein nutrients, probiotics and caffeine was not associated with any personality traits in the athletes. Conclusions: Further relationships of the Big Five personality traits were demonstrated with the use of effective dietary supplements by athletes; the most unambiguous correlations were described for neuroticism and conscientiousness in such a way that the use of isotonic drinks, as well as energy bars and gels, was connected with a low level of neuroticism, while the use of creatine was connected with high conscientiousness.

## 1. Introduction

The basis of proper nutrition for athletes should be a rational, diverse and balanced diet, rich in high-density products [1,2]. The correct nutrition of athletes is a key factor determining health and exercise skills, as well as effective post-performance recuperation. It provides an optimal supply of energy in addition to building and regulatory substrates that control metabolic processes and promote the regulation of water and electrolytes. It further facilitates acid–base management, while maintaining the body’s proper antioxidant status [3,4,5,6,7]. Meta-analytical studies allow for indications of food’s potentially ergogenic effects, including those of beetroot fruit and juice [8,9].

Rational nutrition in athletes can be enriched with supplements, which are an additional source of energy. They also build and regulate substrates, and these stimulate energy generation, mass development and muscle strength, while improving physical performance [10,11,12]. The standard in the use of dietary supplements in sports is determined by recommendations prepared by the Australian Sports Institute (AIS) and the International Olympic Committee (IOC). The classification of supplements for groups A, B, C and D takes scientific evidence as well as other aspects determining safety, legality and effectiveness in improving sports results into account. According to the latest AIS classification (from March 2021), in group A (agents with scientifically confirmed effectiveness and safety of use), there are three types of nutrition for sportspersons: special foods for athletes (beverages, electrolyte supplements, energy gels, protein nutrients, etc.), medical supplements (for athletes with deficiencies, including iron, calcium, zinc, vitamin D, multivitamins and probiotics) and ergogenic aids, which improve physical performance (caffeine, beta-alanine, sodium bicarbonate, beetroot/nitrate juice, creatine and glycerol) [10,13,14,15]. Experts suggest that both foods providing a special purpose for athletes and dietary supplements should only be used if there is strong evidence confirming their effectiveness, legality and safety of use, but these can also be used under conditions of rigorous competition [14,15].

The nutritional behaviour of athletes is dynamic and conditioned by numerous factors, including environmental and individual ones [16,17,18]. An important area of nutritional behaviour conditions includes psychological factors, i.e., personality traits. Personality is treated as an internal regulation system, allowing for adaptation to selected situations and the environment, as well as an internal integration of thoughts, feelings and behaviours [19]. One of the models regarding the approach and description of personality is the five-factor personality model proposed by Costa and McCrae, which, in the last few decades, has become one of the dominant paradigms in the psychology of personality features [20]. The so-called “Big Five” model proposed by Costa and McCrae [21] consists of five main personality dimensions, namely neuroticism, extraversion, openness to experience, agreeableness and conscientiousness. This set of dimensions from the Big Five allows for multifaceted personality characterisation and interpretation [19,20,21]. In our previous works, the predictive significance of personality traits in athletes’ nutrition was demonstrated. Described, among others, was the relationship of low neuroticism and high conscientiousness with more rational eating behaviours of team sport athletes [22,23].

The literature available on the functional properties of individual supplements, including ergogenic aids, is highly extensive [11,12,24,25,26,27,28,29,30,31,32,33,34,35]. In numerous studies, the use of supplements by athletes of various disciplines is described in detail [34,36,37,38,39,40,41,42,43,44,45]. However, there are fewer research works on the individual conditions of athletes using these measures. The works existing in this area concern, in particular, the relationship between ergogenic agents and personal resources, including a sense of generalised self-efficacy and the optimism that may be present [38,39,40]. On the other hand, the authors of this work were not acquainted with publications on personality conditions regarding the use of dietary supplements by high-performance athletes. The results obtained can be used to individualise the educational and nutritional impact of team sport players. They can further constitute a reference point for research among other groups of athletes, including those from other countries.

Therefore, taking a broad spectrum of factors conditioning nutrition into account, as well as the key importance of diet and proper supplementation for maintaining health and carrying out efforts, the aim of this study was to analyse relationships between personality traits from the five-factor model and the use of selected dietary supplements with scientifically proven effectiveness. This was performed in an elite group of Polish athletes representing high-performance team sports. The following research questions were posed: (1) What is the scale of using selected group A supplements among athletes? (2) How are the relationships between personality traits and the use of selected dietary supplements from group A shaped among athletes (by assessing the level of personality traits in groups of persons using and not applying individual groups of agents)? A research hypothesis was adopted stating that low neuroticism and high conscientiousness favour the use of effective dietary supplements by athletes. 

## 2. Material and Methods 

### 2.1. Participants 

This study was conducted in a group of 213 Polish athletes (men) engaging in competitive team sports, including basketball (*n* = 54), volleyball (*n* = 53), football (*n* = 53) and handball (*n* = 53). The basic criterion for inclusion in the study group was playing sports at a competitive level at the highest class level in Poland for at least 3 years. Other inclusion criteria were as follows: age of majority, good health and regular training during the research period. The basic exclusion criteria were belonging to the lower league class, failure to meet the criterion of minimum sports experience (3 years) and health condition preventing regular participation in training during the study period. The evaluated athletes, in relation to the current classification determining level of activity and sport abilities [46], can be assigned to Tier 3 (Highly Trained/National Level).

The research was conducted at 50 sports clubs (18 basketball clubs, 19 volleyball, 13 football and 10 handball clubs). The study included athletes aged 18–36 (M = 26.1; SD = 4.5), with sports experience ranging from 3 to 20 years (M = 8.2; SD = 4.5). The trial was carried out in accordance with the principles of the 1964 Helsinki Declaration, and the participants’ written informed consent was obtained before the trial was conducted. The research protocol was approved by the Bioethics Committee of the District Medical Chamber in Kraków (No. 105/KBL/OIL/2021).

### 2.2. Instruments

To assess the use of dietary supplements by athletes, a proprietary questionnaire was implemented. This included a list of supplements with a three-stage response scale (1, from ‘never’; 2, ‘periodically, during pre-start and/or starting periods’; and 3, ‘regularly—during the entire annual training cycle’). The list of agents included multivitamin and multimineral preparations, protein, carbohydrate and protein-carbohydrate nutrients, isotonic drinks, electrolytes, carbohydrate gels and bars, branch chain amino acids, creatine, β-Hydroxy-β-methylbutyric acid (HMB), Omega 3 polyunsaturated fatty acids, anti-oxidants, caffeine, probiotics and joint-protection preparations. The questionnaire was subjected to a validation procedure. The relevance of the test was estimated using the method of re-testing (*n* = 32). The value of the signed-rank correlation coefficient was calculated, and the zero hypothesis test H_0_:R = 0 was performed via the Student’s *t*-test, with a result confirming the reliability of the scale (*r* = 0.385; *p* = 0.035). Successful internal compatibility was also confirmed (Cronbach’s α factor totalled 0.78). For time-related reasons (the period of implementing the study vs. time for preparing the publication), the current classification of supplements (according to the AIS from March 2021) was not taken into account in the list of supplements included in the questionnaire. Selected supplements with scientifically confirmed effectiveness (from group A according to the latest AIS classification) were included in the work.

The Polish adaptation of the NEO-PI-R inventory (Neuroticism–Extraversion–Openness Personality Inventory—Revised), by P.T. Costa and R.R. McCrae [21], was used [47]. The NEO-PI-R includes 240 statements assessed by the respondent on a five-stage scale (from ‘I completely disagree’ to ‘I completely agree’). The claims relate to five personality factors (scales), and there are six sub-scales related to them. The reliability of measuring five dimensions in the Polish adaptation of the questionnaire is appropriate, with values of 0.81 (agreeableness), 0.85 (extraversion and conscientiousness) and 0.86 (neuroticism and openness to experience) [47]. In the present work, psychological analyses were limited to the five basic personality dimensions of athletes. Personality features regarding the examined group of athletes have already been the subject of research in our other publication [48]; therefore, they will not be given in this work.

### 2.3. Statistical Analysis 

The collected numerical material was subjected to statistical analysis using the Statistica 13.3 package. The values obtained for the basic measures of the position and dispersion concerning the values of personality features (Neuroticism, Extraversion, Openness, Agreeableness, Conscientiousness) and the percentage of individual categories regarding the frequency of supplement use were calculated. Differentiation in the level of personality traits (Neuroticism, Extraversion, Openness, Agreeableness, Conscientiousness scales) was tested depending on the qualification of respondents in response to a category denoting frequency of supplement use (‘never’, ‘periodically’, ‘regularly’). Due to the variables not being normally distributed, non-parametric analyses were used. The non-parametric Kruskal–Wallis test for a single-factor system was used in the analyses (along with Dunn’s post hoc test with Bonferroni’s correction), as well as the non-parametric Mann–Whitney test (comparison of two groups), because it was planned to determine differences between groups with varying supplement consumption frequencies. The analysis was carried out with the level of significance adopted as α = 0.05 and the test probability of *p* ≤ 0.05. Interpretation concerning the direction of differentiation between the categories demonstrating the frequency of supplement use was conducted on the basis of moderate-ranking values.

## 3. Results 

### 3.1. Use of Dietary Supplements by Athletes

The surveyed participants, in the largest percentage (90.1%), chose isotonic drinks (including 65.3% regularly during the entire annual training cycle) from among the proposed special foods for athletes. To a high extent (over 80%), they used protein (usually periodically) and carbohydrate gels and bars (usually regularly in the entire annual training cycle). With regard to so-called medical supplements, the athletes, in the largest percentage (79.8%), chose multimineral (usually regularly) and multivitamin preparations (66.7%, usually periodically). To a lesser extent, they reached for probiotics (42.3%, usually periodically). Among the ergogenic aids, in the largest percentage, they chose creatine (69.5%, usually periodically) and caffeine (53.1%, usually periodically), and they reached for bicarbonates the least often (14.6%) (Table 1).

### 3.2. Relationships between Personality Traits and the Use of Dietary Supplements by Athletes

It was shown that athletes using isotonic drinks (periodically or regularly) were characterised by a lower level of neuroticism than other competitors (*p* = 0.030). At the same time, athletes who consumed isotonic drinks periodically were characterised by the lowest levels of openness, and those who consumed them always were characterised by the highest levels of openness (*p* = 0.025). Competitors consuming energy bars and gels (periodically or regularly) were characterised by a lower level of neuroticism than those who did not use these agents (*p* = 0.002). Furthermore, athletes who periodically consumed these gels and bars were characterised by the lowest levels of agreeableness, and those who never consumed them were characterised by the highest levels of agreeableness (*p* = 0.013). Athletes periodically taking multivitamin preparations were characterised by a lower level of extraversion (*p* = 0.042) and openness compared to the other participants (*p* = 0.050). Athletes periodically using multimineral preparations were characterised by a higher level of agreeableness than the others (*p* = 0.025). It was shown that athletes periodically using sodium bicarbonate were characterised by a higher level of extraversion (*p* = 0.009) and a lower level of agreeableness (*p* = 0.050) than the remaining subjects. Athletes who did not consume creatine were characterised by the lowest level of conscientiousness among all participants (*p* = 0.013). The use of protein nutrients, probiotics and caffeine was not related to the personality traits of the athletes (*p* > 0.05) (Table 2).

## 4. Discussion 

This study allowed us to show a diverse scale of using group A dietary supplements (according to the current AIS classification) and significant relationships between specific personality traits and the use of selected supplements by high-class Polish athletes in high-performance team sports.

When we discuss how supplements are disseminated in the group under study, it should be noted that among all agents, the participants most often reached for food specially designed for athletes, including isotonic drinks, energy bars and gels (usually regularly) as well as protein nutrients (usually periodically). They also frequently took medical supplements which are recommended for nutritional deficiencies, including mineral and vitamin preparations (with recommended ingredients, i.e., Ca, Fe, Zn and vitamin D) and ergogenic aids, which allow an increase in physical performance (creatine and caffeine). To a lesser extent, however, they reached for probiotics (from the group of medical supplements), and to the lowest degree, they reached for bicarbonates (from the group of ergogenic agents).

The high prevalence of isotonic drink consumption is justified by its significance in effective hydration of the body, and also maintaining water as well as electrolyte balance in conditions of physical effort, while accelerating post-workout glycogen resynthesis [34,49,50,51,52,53]. Gel (65–70% carbohydrates) and carbohydrate bars used by the studied athletes were justified in the context of their significance for the rapid replenishment and levelling of energy reserves and losses. This could be conducive to effective muscle work, delaying fatigue of the central nervous system and intensifying the pace of post-performance recuperation. In turn, the intake of protein nutrients as a source of building ingredients, in a condensed and well-absorbed form, could be conducive to effectively balancing cell protein losses and post-workout muscle regeneration [27,34]. Also, the frequent intake of vitamin and mineral supplements was important, given the role of the components in regulating the maintenance and restoration of the body’s homeostasis in conditions of physical exertion [34,49]. Group B vitamins catalyse metabolic processes. Antioxidant vitamins and polyphenols increase the antioxidant potential of cells while providing protection against the effects of intensified oxidative stress which tends to develop in athletes. Mineral salt supplementation could have a significant impact on the regulation of water, electrolyte and acid–base management as well as neuromuscular excitability and haematopoietic processes [34,49]. The significance of vitamins D and E for well-being and regeneration was confirmed among basketball players [54]. The use of carbohydrate supplements as well as vitamins and mineral salts could also be justified with regard to the results of numerous studies in the scope of quantitative dietary assessment, indicating a low supply of various nutrients in the diet of athletes, including energy, carbohydrates, mineral salts (inter alia, K and Ca) and vitamins (group B and vitamin D) [55,56,57]. Ergogenic agents as stimulators of psychophysical performance (i.e., caffeine) affect the delay of central nervous system fatigue and the release of free fatty acids. Creatine has an effect on muscle structure and strength, while stimulating energy generation during vigorous physical exercise. This increases physical performance and extends the time of effective physical exertion [25,26,28,30,31,32,33,34]. On the other hand, the scale of probiotic use was low. Agents that have a positive effect on the condition of the intestinal microbiota prevent dysbiosis, which implies numerous gastric and systemic disorders lowering the effectiveness of sport training. A meta-analysis confirmed that psychological stress in athletes intensifies intestinal microbiota disorders and increases dysbiosis health risks [58,59,60,61]. The use of probiotics, in connection with their positive impact on the composition of the body, reduction of cortisol and lactate levels, and increase in the synthesis of neurotransmitters regulating cognitive functions and mood, could have a positive impact on the functioning of athletes [62]. The lowest was the scale of bicarbonate usage, which corresponds to the physiology of effort in team sports [54].

In this study, among team sport players, it was shown that the use of various ergogenic substance types was conducive to accelerating regeneration processes (muscles, rebuilding glycogen, eradicating fatigue and improving immunity), which made it easier to prepare for subsequent training sessions/competitions. In this group of agents, carbohydrates, proteins, liquids, creatine, Omega 3 polyunsaturated fatty acids, vitamin D and antioxidants were indicated [5]. A systematic review showed a positive impact of polyphenol consumption on muscle regeneration after physical exertion. A dose of 60 mL/day, taken twice a day for at least 7 days, reduced muscle soreness in team sport athletes [35]. Also, in a new systematic review regarding the impact of selected dietary supplements on the sports results of elite players, the importance of caffeine, creatine, protein nutrients and isotonic drinks as well as juice from tart cherry and beetroot concentrates for exercise abilities was confirmed [34].

The use of agents to support exercise abilities and the pace of post-performance recuperation in athletes was also confirmed by other authors who pointed to their broad dissemination in elite sport (from 40 to 100% of competitors, depending on the type of sport, sport level and supplement definition) [63]. The use of these substances is described in competitive handball players (most often referring to drinks for athletes, energy bars and caffeine-containing products) [41]. Their usage is also discussed with regard to individual and team sport disciplines (most often among players of individual disciplines representing at a high sports level) [34], among elite Spanish athletes (usually BCAA proteins and amino acids, on a varied scale depending on age, sex, sports level and discipline) [42], among amateur and professional rugby players (usually whey protein, caffeine, sports drinks, energy bars and creatine monohydrates) [43], among Turkish athletes (drinks for sportspersons, magnesium, vitamins C and D, caffeine, sports bars and whey protein, depending on gender and sports levels) [44], among Italian beach volleyball players (vitamins C and B, protein and caffeine) [45], among Polish American football players (usually isotonic drinks, vitamin and mineral preparations and protein nutrients) [38] and among competitive footballers (typically, vitamins, mineral salts, isotonic drinks, protein nutrients and creatine) [39]. 

Before discussing the relationships of personality traits with the use of dietary supplements by athletes, the basic personality characteristics of the studied athletes should be indicated. In this respect, it was found that competitors obtained high results in the areas of extraversion, openness, willingness and contentiousness and low results in the area of neuroticism [48]. A low level of neuroticism among athletes was also described in other studies conducted in a group of competitive athletes, including those training in team sports [64,65], and also among Polish athletes [66,67].

In the present study, relationships were shown between selected personality traits and the use of dietary supplements by athletes. A varied level was found for certain character qualities in groups of athletes using (periodically or regularly) and not using specific agents. However, some of the noted relationships were ambiguous, which concerned, in particular, the dimensions of openness, willingness and extraversion. However, unambiguous relationships were described for the dimensions of neuroticism and conscientiousness. In this respect, it was found that lower neuroticism and higher conscientiousness favoured the use of dietary supplements. In the case of neuroticism, a lower level is described in athletes regularly or periodically consuming isotonic drinks as well as energy gels and bars. In the case of conscientiousness, the lowest level was described among athletes not taking creatine. Due to the key importance of isotonic drinks and energy substrates for exercise abilities, the predictive significance of low neuroticism for the use of supplements from a special food group for athletes is emphasised.

Other studies on the psychological conditions of using dietary supplements by athletes allowed the predictive meaning of specific personal resources, including the sense of generalised self-efficacy and available optimism, to be shown [38,39,40]. In this respect, it was demonstrated that American football players with a lower level of efficacy used multivitamin supplements significantly more often than those characterised by a higher level [38]. This could be related to the fact that athletes with a lower sense of generalised self-efficacy presented less rational nutritional choices, increasing the risk of nutritional deficiencies [68]. In turn, among competitive athletes, it was shown that individuals with a higher level of available optimism showed more rational nutritional choices and more frequent probiotic use [40]. The studies also allowed the confirmation of a relationship between personality traits, diet quality and the nutritional behaviour of athletes, including players training in team sports [22,23,48] and physical education students, also presenting increased levels of physical activity [69]. These studies, despite some ambiguity in their results, also allowed the confirmation of a positive relationship between low neuroticism and a higher level of the pro-healthy diet index. However, with the intensification of neuroticism (and its sub-scales: anxiety, depression, hostility and shyness), the health-promoting quality of the diet decreased [48]. The elite group of Polish athletes training in team sports also indicated the positive predictive significance of conscientiousness with regard to more rational nutritional behaviour, including regular consumption of meals and dairy products, as well as limiting sweet and salty snacks [22]. Among the high-class team sport players, the negative predictive significance of neuroticism and agreeableness for the quality of peri-exercise nutritional behaviour was found [23]. In the present study, ambiguity regarding the results related to the use of dietary supplements depending on other dimensions of the Big Five model (extraversion, openness and agreeableness) corresponds to the ambiguity of relationships between these personality traits and diet health quality, as well as nutritional behaviours of athletes examined in our previously published works [22,48]. Therefore, it can be stated that the low level of neuroticism, associated, for example, with emotional stability, and a high level of conscientiousness, connected with, for example, the ability to control stimuli and focus on achieving specific goals [19], promote the use of dietary supplements with proven effectiveness and safety of use. Thus, it may be assumed that low neuroticism and high conscientiousness may increase tendencies towards more proper nutritional and supplementation behaviours, which can have a positive impact on athletes. Of course, research would be needed to cover not only aspects of psychology and sports dietetics, but also the physiology of physical efforts.

The limitations of the work are primarily related to the self-described nature of the applied research tools, with failure to take all supplements included in the list of effective supplements into account (according to the AIS from 2021). Furthermore, demographic and sports variables (age, competitive experience and discipline) were also not considered. Another significant limitation of the work is its exclusive focus on men and the failure to include women. Other potential confounding factors were also not taken into account, including those related to various aspects of lifestyle and health (i.e., diet, nutritional status and sleep quality), socio-economic status, etc. These indicated restrictions, as well as others, may set the direction of further work, the purpose and subject of which should be a comprehensive assessment of personality conditions related to various diet areas (also in quantity) and supplementation among athletes, taking sex, sport experience, sport level and type of discipline into account. Subsequent studies could focus on personality conditions in consideration of the quantitative aspects of athletes’ diets (therefore, energy consumption, macronutrients, vitamins and mineral salts), which would be a contribution to the comprehensive assessment of diets among athletes. They could also focus on the relationships existing between the personality traits, diet and exercise capacity of athletes. Future research in this area could also be undertaken to address diverse population groups and different sporting contexts.

## 5. Conclusions

1. Among the athletes training in team sports, effective dietary supplements were demonstrated. These included special foods for athletes (especially isotonic drinks, protein nutrients, gel and carbohydrate bars), medical supplements (including multimineral and multivitamin preparations) and ergogenic aids (especially creatine and caffeine).
2. Significant relationships between the Big Five personality traits and the use of effective dietary supplements by athletes were noted, with unambiguous relationships described for the dimensions of neuroticism and conscientiousness (a lower level of neuroticism was associated with the consumption of isotonic drinks, energy gels and bars, while a higher level of conscientiousness concerned athletes who took creatine). Therefore, it can be assumed that low neuroticism and high conscientiousness, associated with the use of some effective dietary supplements, may contribute to improving the exercise capacity of athletes.
3. There is a need for further research in order to explain the mechanisms of the relationships observed between athletes’ personalities and diets. The personality traits of athletes should be taken into account in dietary education.

## Figures and Tables

**Table 1:** Use of selected group A dietary supplements by athletes training in team sports (% of indications).

Selected Group A Supplements		Never	Periodically (During the Pre-Start and/or Starting Period)	Regularly (During the Year-Long Training Cycle)	Total (Periodically and Regularly)
Special foods for athletes	Isotonic drinks	9.9	24.9	65.3	90.1
	Energy gels and bars	19.2	37.1	43.7	80.8
	Protein supplements	15.5	44.1	40.4	84.5
Medical supplements	Multivitamins (with vitamin D)	33.3	37.1	29.6	66.7
	Multiminerals (with Fe, Ca, Zn)	20.2	33.8	46.0	79.8
	Probiotics	57.7	27.7	14.6	42.3
Ergogenic aids	Caffeine	46.9	46.9	6.2	53.1
	Sodium bicarbonate	85.4	14.6	0.0	14.6
	Creatine	30.5	61.5	8.0	69.5

**Table 2:** Differences in the level of personality traits depending on the use of selected dietary supplements (special-purpose foods for athletes, medical supplements and ergogenic aids) by athletes (results of the Kruskal–Wallis and Mann–Whitney tests).

Supplements	Usage	N	E	O	A	C
		Moderate Rank				
Isotonic drinks	Regularly	101.4a	106.4	115.1b	107.9	109.7
	Periodically	108.8a	99.8	88.9a	105.6	108.2
	Never	139.3b	128.8	98.9ab	104.7	86.3
		*p* = 0.030	*p* = 0.184	*p* = 0.025	*p* = 0.958	*p* = 0.265
Carbohydrate gels/bars	Regularly	98.0a	98.3	103.5	110.0ab	112.2
	Periodically	102.1a	113.7	111.5	93.1a	107.6
	Never	136.7b	113.7	106.3	127.0b	94.0
		*p* = 0.002	*p* = 0.194	*p* = 0.694	*p* = 0.013	*p* = 0.284
Protein supplements	Regularly	108.4	107.0	109.9	114.5	110.7
	Periodically	108.0	107.8	109.7	105.8	99.7
	Never	100.7	104.7	91.6	90.8	117.9
		*p* = 0.811	*p* = 0.969	*p* = 0.293	*p* = 0.164	*p* = 0.263
Multivitamins	Regularly	102.9	126.2b	125.4b	103.6	109.7
	Periodically	111.5	90.4a	94.6a	115.0	102.9
	Never	105.6	107.0ab	101.5ab	101.1	109.1
		*p* = 0.692	*p* = 0.042	*p* = 0.050	*p* = 0.336	*p* = 0.757
Multiminerals	Regularly	103.3	108.9	113.9	98.6a	118.2
	Periodically	110.1	96.5	102.2	122.9b	104.0
	Never	106.4	113.9	99.3	99.4a	104.3
		*p* = 0.842	*p* = 0.187	*p* = 0.307	*p* = 0.025	*p* = 0.411
Probiotics	Regularly	108.9	95.7	99.0	107.6	112.3
	Periodically	105.6	113.8	105.3	104.4	101.2
	Never	107.2	106.6	109.8	108.1	108.5
		*p* = 0.970	*p* = 0.410	*p* = 0.660	*p* = 0.931	*p* = 0.661
Caffeine	Regularly	129.5	110.5	120.2	103.0	127.1
	Periodically	106.7	107.9	113.0	109.2	104.6
	Never	104.4	105.7	99.3	105.3	106.8
		*p* = 0.382	*p* = 0.948	*p* = 0.212	*p* = 0.876	*p* = 0.461
Sodium bicarbonate	Periodically	112.0	133.8	108.2	87.1	112.2
	Never	106.2	102.4	106.8	110.4	106.1
		*p* = 0.630	*p* = 0.009	*p* = 0.907	*p* = 0.050	*p* = 0.612
Creatine	Regularly	94.4	118.2	107.5	95.8	139.4b
	Periodically	106.0	106.9	111.0	105.1	109.3ab
	Never	110.7	102.6	96.9	112.3	92.1a
		*p* = 0.618	*p* = 0.640	*p* = 0.318	*p* = 0.560	*p* = 0.013

Identical letter symbols next to the mean rank values indicate no significant differences between means for Dunn’s test (α = 0.05). N—Neuroticism, E—Extraversion, O—Openness, A—Agreeableness, C—Conscientiousness.

## Data Availability

Data are contained within the article.
